# Surveying the interest of individuals with upper limb loss in novel prosthetic control techniques

**DOI:** 10.1186/s12984-015-0044-2

**Published:** 2015-06-13

**Authors:** Susannah M. Engdahl, Breanne P. Christie, Brian Kelly, Alicia Davis, Cynthia A. Chestek, Deanna H. Gates

**Affiliations:** Department of Biomedical Engineering, University of Michigan, Ann Arbor, MI USA; Department of Physical Medicine and Rehabilitation, University of Michigan, Ann Arbor, MI USA; University of Michigan Orthotics and Prosthetics Center, Ann Arbor, MI USA; Neurosciences Program, University of Michigan, Ann Arbor, MI USA; Department of Electrical Engineering and Computer Science, University of Michigan, Ann Arbor, MI USA; School of Kinesiology, University of Michigan, Ann Arbor, MI USA

**Keywords:** Survey, Upper limb loss, Upper limb prostheses, Prosthetic design, Prosthetic control

## Abstract

**Background:**

Novel techniques for the control of upper limb prostheses may allow users to operate more complex prostheses than those that are currently available. Because many of these techniques are surgically invasive, it is important to understand whether individuals with upper limb loss would accept the associated risks in order to use a prosthesis.

**Methods:**

An online survey of individuals with upper limb loss was conducted. Participants read descriptions of four prosthetic control techniques. One technique was noninvasive (myoelectric) and three were invasive (targeted muscle reinnervation, peripheral nerve interfaces, cortical interfaces). Participants rated how likely they were to try each technique if it offered each of six different functional features. They also rated their general interest in each of the six features. A two-way repeated measures analysis of variance with Greenhouse-Geisser corrections was used to examine the effect of the technique type and feature on participants’ interest in each technique.

**Results:**

Responses from 104 individuals were analyzed. Many participants were interested in trying the techniques – 83 % responded positively toward myoelectric control, 63 % toward targeted muscle reinnervation, 68 % toward peripheral nerve interfaces, and 39 % toward cortical interfaces. Common concerns about myoelectric control were weight, cost, durability, and difficulty of use, while the most common concern about the invasive techniques was surgical risk. Participants expressed greatest interest in basic prosthesis features (e.g., opening and closing the hand slowly), as opposed to advanced features like fine motor control and touch sensation.

**Conclusions:**

The results of these investigations may be used to inform the development of future prosthetic technologies that are appealing to individuals with upper limb loss.

**Electronic supplementary material:**

The online version of this article (doi:10.1186/s12984-015-0044-2) contains supplementary material, which is available to authorized users.

## Introduction

In 2005, there were approximately 41,000 people in the U.S. living with major upper limb loss, with a projected increase of 131 % by 2050 [1]. Although upper limb loss has been found to be considerably more life-altering than lower limb loss [2], there are few commercially available options for upper limb prostheses. Generally, individuals with upper limb loss must choose between passive, body-powered, and myoelectric prostheses. Passive prostheses provide an aesthetically-pleasing substitute for the missing limb but do not offer functional movement. Body-powered prostheses use a harness system to capture movements of the residual limb that actuate a terminal device. Myoelectric prostheses are controlled by surface electromyography signals recorded from the muscles of the residual limb. All of these devices are limited in their ability to provide multiple degrees of freedom, sensory feedback, and quick, smooth movements.

Perhaps unsurprisingly, it is common for individuals with upper limb loss to abandon prosthesis use. Mean estimates of prosthesis rejection rates are 26 % for body-powered prostheses and 23 % for electric prostheses [3]. However, 74 % of non-users report willingness to reconsider using a prosthesis if improvements in technology are made at a reasonable cost [4]. Some of the previously reported priorities for device improvement include increased range of movement (especially at the wrist), coordinated movement of multiple joints, adaptability of grip strength, greater intuitiveness of control, and increased sensory feedback [4–6].

One significant impediment to the development of multi-degree of freedom upper limb prostheses is the difficulty of controlling these devices. Although myoelectric control is the current state of the art [7], the functionality of a myoelectric prosthesis is limited by the number of independent electromyographic signals that can be recorded from the residual limb. Even in individuals with transradial limb loss, it is generally possible to identify only two independent recording sites [7]. One possible way to avoid this problem is through pattern recognition, in which specific signal characteristics are extracted and used to control a prosthesis. Examples of pattern recognition techniques include fuzzy logic classifiers [8], neural networks [9], blind source separation [10], and supervised adaptive paradigms [11]. Myoelectric control has also been used in conjunction with inertial measurement units placed on the foot to control a multi-degree of freedom prosthesis [12]. Other examples of foot control methods include sensorized insoles worn inside the shoe [13] and lower extremity “stockings” with integrated goniometers [14]. Alternative control methods based on myoacoustic (vibration associated with muscle contraction) [15], myopneumatic (pressure distributions associated with muscle contraction) [16], and myokinemetric (change in muscle position during contraction) [17] signals have also been proposed.

Other techniques for prosthesis control are more invasive. For example, targeted muscle reinnervation involves transferring residual peripheral nerves to specific muscles near the amputated limb, thereby creating additional independent electromyographic signal sites that may be used to control a prosthesis [18]. Implantable myoelectric sensors are chronically implanted into residual muscles via minimally invasive surgical techniques [19, 20]. Each sensor records at the source of muscle contraction and detects only one channel of an electromyographic signal, so implanting many of these devices throughout the limb could allow for more than two simultaneous degrees of freedom [21]. Additionally, implantable cuff electrodes interface with peripheral nerve axon populations, which remain viable after limb amputation. For example, a flat interface nerve electrode reshapes the nerve into a flat configuration, bringing the central axon populations to the surface [22]. Flat interface nerve electrodes have been used in animals and humans to record and stimulate different populations of neurons based on the intent to move or experience sensation [22, 23]. A more invasive technique for recording sensory and motor information in the peripheral nerves involves penetrating Utah slanted microelectrode arrays [24]. Similarly, thin-film longitudinal intra-fascicular electrodes involve a set of contacts spaced at fixed, predetermined distances on a single flexible substrate that can be threaded into a nerve [25]. Because these devices penetrate the nerve, they offer a higher degree of specificity and signal-to-noise ratio. The most invasive technique for recording sensory and motor command signals involves the use of multi-electrode arrays that penetrate 1–2 mm into the cortex. Past studies have demonstrated that individuals with paralysis can use signals from the brain to control a robotic arm for self-feeding [26, 27].

Despite the great diversity among current research efforts, we will group these prosthesis control techniques into four categories: myoelectric control, targeted muscle reinnervation, peripheral nerve interfaces, and cortical interfaces. These categories are intended to describe general *types* of technique, rather than any specific approach. For the purposes of this paper, the “peripheral nerve interfaces” category refers to electrodes implanted in the residual limb to record neural signals directly from the peripheral nervous system. The “cortical interfaces” category refers to electrode arrays implanted in the brain to record action potentials directly from motor neurons. Thus, one of the four categories is noninvasive (myoelectric control) and the remaining three are invasive (targeted muscle reinnervation, peripheral nerve interfaces, and cortical interfaces).

Although targeted muscle reinnervation, peripheral nerve interfaces, and cortical interfaces have the potential to increase the functionality of upper limb prostheses, they have increased surgical risk compared to noninvasive techniques. It is important to understand whether individuals with upper limb loss are willing to accept these risks if they could have a more functional prosthesis. This information could help refine or redirect research efforts towards developing prostheses that are appealing for individuals with upper limb loss.

In this study, we used an online survey to evaluate general interest in noninvasive and invasive prosthetic control techniques. We proposed the following hypotheses: (1) Individuals will be more willing to try noninvasive than invasive techniques. (2) Individuals will be more willing to try invasive techniques that offer higher levels of functionality.

## Methods

### Survey design

An anonymous online survey was designed using Qualtrics Research Suite (Qualtrics, Provo, UT) to explore the opinions of individuals with upper limb loss regarding prosthetic control techniques. Several local researchers, clinicians and individuals with upper limb loss contributed to the development and pilot testing of the survey.

The survey required approximately 15–30 minutes to complete. It included both closed and open-ended questions regarding basic demographics, prosthesis usage, satisfaction with functional abilities, prosthesis design priorities, and interest in prosthetic control techniques. The survey was customized for each participant based on his or her responses to the demographics and prosthesis usage questions. For example, participants with congenital limb loss were not asked about their occupation at the time of amputation. Participants were required to answer every question that was presented. Early versions of the survey did not force participants to answer every presented question, so several questions do not have responses from every participant who viewed the question.

**Fig. 1:**
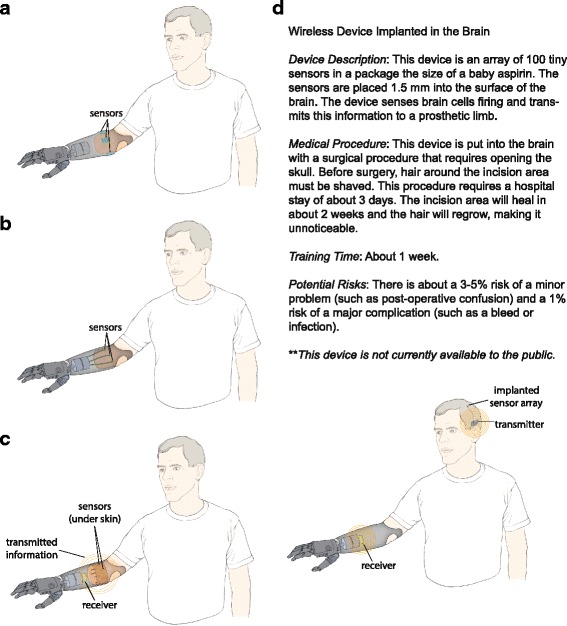
Drawings of each technique. Participants viewed drawings for myoelectric control (**a**), targeted muscle reinnervation (**b**), peripheral nerve interfaces (**c**), and cortical interfaces (**d**). Brief explanations were included with each drawing. (All drawings and explanations are included in Additional file 1)

In the final section of the survey, participants were asked about their interest in myoelectric control, targeted muscle reinnervation, peripheral nerve interfaces, and cortical interfaces. Participants viewed a simple drawing and a brief explanation of each technique, along with a description of any associated medical procedures and potential risks (Fig. 1). Participants were then asked to rate their likelihood of using the interface at each of six levels of performance, which were roughly ordered from basic to advanced (Table 1). For example, the most basic feature was opening and closing the hand slowly. Each successive level became more advanced, culminating with touch sensation in the missing limb. The questions were phrased using the following syntax: “With the procedures and risks in mind, how likely are you to have the device if it could let you * insert specific feature* *?*” Responses were collected on a 5-point Likert scale from “very unlikely” to “very likely.” Participants were also given the option to provide open-ended comments regarding each technique.

**Table 1:** Comparison of discrete and cumulative wording versions

Performance Level	Discrete	Cumulative
1	Open and close your hand slowly	Open and close your hand slowly
2	Open and close your hand, and also rotate your wrist	Do all the above AND rotate your wrist
3	Move to any location in your workspace and perform a simple grasp	Do all the above AND move to any location in your workspace and perform a simple grasp
4	Move to any location in your workspace and perform one of several types of grasps, in which you can control the amount of force used	Do all the above AND perform one of several types of grasps, in which you can control the amount of force used
5	Perform fine tasks like writing with a pen or typing	Do all the above AND perform tasks that require fine motor control (such as writing with a pen or typing)
6	Perform fine tasks and have touch sensation in the missing limb	Do all the above AND have touch sensation in the missing limb

Participants were also asked to rate how important each level of performance was to them. This question was presented independently of specific techniques and was intended to gauge the participants’ general interest in each feature. Responses were collected on a 5-point Likert scale from “very unimportant” to “very important”.

We used two different wording versions for the levels of performance (Table 1). The wording was initially intended to present each of the six levels as cumulative, where each level expanded on the previous ones. This wording was chosen under the assumption that participants would only be interested in the invasive prosthetic control techniques if they could offer high levels of functionality. With cumulative wording, the more advanced levels would combine several different features and thus might be more appealing than a single feature. However, inspection of the responses from the first 35 participants suggested that they did not respond as anticipated. After reviewing the wording, we realized that the phrasing was somewhat misleading and may not actually have been interpreted as cumulative. The wording was then changed to be explicitly cumulative in order to align with our original intentions. All subsequent participants viewed this version. For the remainder of this paper, the initial wording will be referred to as “discrete” and the alternate wording will be referred to as “cumulative”. A more detailed discussion of these issues will be presented later.

### Survey distribution and data collection

All individuals over age 18 with major upper limb loss (above partial hand level) were invited to participate. The survey was administered via tablet computer to patients at the University of Michigan Orthotics and Prosthetics Center. A link to the survey was posted online through various forums and mailing lists (e.g., AMP-L, OANDP-L, Arm-Amp, I-CAN, Amputee Empowerment Partners, Eastern Amputee Golf Association, amputeeforums.com, healthboards.com, Facebook groups). Flyers promoting the survey were distributed by clinicians and researchers at several institutions within the United States. Flyers were also distributed to attendees at a local educational event for individuals with limb loss. This study was granted exempt status by the Institutional Review Board at the University of Michigan.

### Data analysis

Statistical analyses were performed using SPSS 21 (IBM, Armonk, NY). Frequency distributions were used to investigate the demographic characteristics of the participants. A two-way repeated measures analysis of variance with Greenhouse-Geisser corrections was used to examine the effect of technique type and performance level on participants’ interest in each technique. Pairwise comparisons were performed using Fisher’s Least Significant Difference method.

## Results

A total of 149 individuals participated in the online survey. Thirty-eight responses were discarded because the participant was under 18 years old (*n* = 2), had only partial hand amputations (*n* = 4), or submitted an incomplete response (*n* = 32). Additionally, the seven responses received during piloting were not included because substantial changes were made to the survey during this time. Thirty-five of the remaining 104 responses used the discrete wording for the levels of performance and 69 used the cumulative wording.

### Demographics

The mean age of the 104 participants was 47 ± 15 years (range: 19–82 years). Seventy participants (67 %) were male and 34 participants (33 %) were female. Ninety-five participants (92 %) had unilateral limb loss. Limb loss most commonly occurred at the transradial and transhumeral levels (Table 2). Trauma was the most common reason for limb loss (65 %), followed by congenital deficiencies (19 %). Among the participants with non-congenital limb loss, the mean age at amputation was 35 ± 15 years (range: 5–69 years). Forty-one participants (48 %) with unilateral, non-congenital limb loss were affected on their dominant arm. Educational attainment levels were diverse, with 98 % of the participants having obtained a high school degree (Table 3).

**Table 2:** Number of amputations at each level

Amputation Level	Unilateral	Bilateral
Forequarter	3	2
Shoulder disarticulation	6	2
Transhumeral	28	3
Elbow disarticulation	5	0
Transradial	42	8
Wrist disarticulation	11	3

**Table 3:** Educational attainment levels

Education Level	Survey^a^	National Averages^a^ ^b^
High school graduate	98 %	88 %
Some college	68 %	58 %
Associate and/or Bachelor’s degree	49 %	41 %
Bachelor’s degree	36 %	32 %
Master’s and/or Doctorate and/or professional degree	21 %	13 %
Doctorate and/or professional degree	5 %	3 %
Doctorate	1 %	2 %

^a^The percentages are cumulative and thus add to over 100 %. For example, it is assumed that all individuals with a Bachelor’s degree also earned a high school degree

^b^Values obtained from [32]

### Prosthesis use and satisfaction

Seventy-two participants (69 %) reported currently using an upper limb prosthesis (at any level of frequency), while only 55 participants (53 %) reported that a prosthesis is “necessary” or “very necessary” in their everyday life. Forty-six of the current prosthesis users (44 %) reported being “satisfied” or “very satisfied” with their functional abilities. Only 14 of the participants (14 %) who do not use a prosthesis reported satisfaction with their functional abilities.

### Overall interest in prosthetic control techniques

**Fig. 2:**
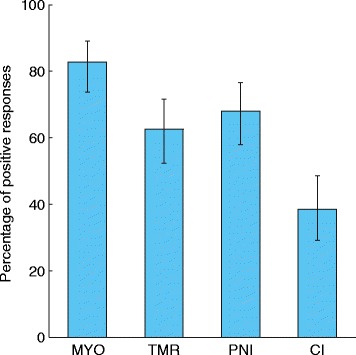
Percentage of positive responses for each technique. A participant’s response was considered positive if they indicated that they would be “likely” or “very likely” to try the technique at any of the six levels of performance. Error bars represent 95 % confidence intervals. (MYO = myoelectric control, TMR = targeted muscle reinnervation, PNI = peripheral nerve interface, CI = cortical interface)

When the responses were collapsed across all six levels of performance, participants expressed the greatest interest in myoelectric control and the least interest in cortical interfaces. Out of all 104 participants, 83 % responded positively to the myoelectric control (Fig. 2). Only 39 % responded positively to the cortical interfaces, while targeted muscle reinnervation and peripheral nerve interfaces were roughly equivalent (63 and 68 %, respectively).

### Interest as a function of technique

**Fig. 3:**
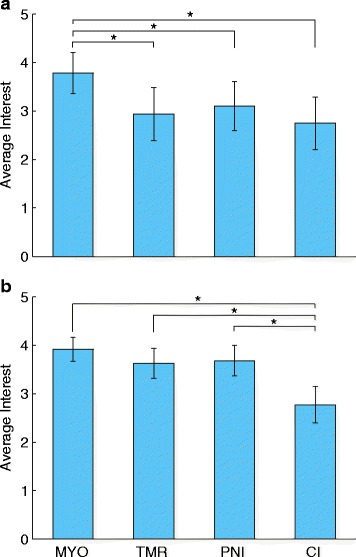
Average responses for each technique. The average Likert score for each technique is shown separately for the discrete (**a**) and cumulative (**b**) wording. *Error bars* represent 95 % confidence intervals. (MYO = myoelectric control, TMR = targeted muscle reinnervation, PNI = peripheral nerve interface, CI = cortical interface)

There was a significant difference in participant interest between the prosthetic control techniques. This was true for both the discrete (*p* < 0.001) and cumulative (*p* < 0.001) wording (Fig. 3). For the discrete wording, participants were significantly more interested in myoelectric control than targeted muscle reinnervation (*p* < 0.001), peripheral nerve interfaces (*p* = 0.001), and cortical interfaces (*p* < 0.001). For the cumulative wording, participants were significantly less interested in cortical interfaces than myoelectric control (*p* < 0.001), targeted muscle reinnervation (*p* < 0.001), and peripheral nerve interfaces (*p* < 0.001).

### Interest as function of performance level

**Fig. 4:**
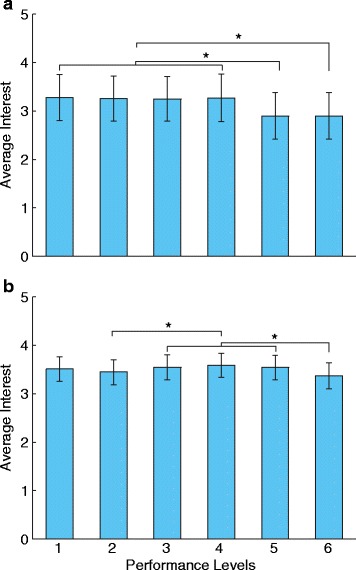
Average responses for each level of performance. The average Likert score for each level of performance is shown separately for the discrete (**a**) and cumulative (**b**) wording. The levels are labeled 1–6 for clarity. (Refer to Table 1 for the exact wording of each level). *Error bars* represent 95 % confidence intervals

We anticipated that participants would be more likely to try each prosthetic control technique if it offered higher levels of functionality. For example, the responses for the most basic level might be “very unlikely” or “unlikely” but increase to “likely” or “very likely” for the most advanced level. However, the initial responses did not follow this expected trend – participants generally responded less positively to more advanced performance levels (Fig. 4a). The wording was then changed to be explicitly cumulative to determine if this observed trend was the result of ambiguous wording or misinterpretation by the participants. A similar trend remained with the cumulative wording – participants generally responded less positively to more advanced performance levels (Fig. 4b).

There were significant differences in participants’ interest in different performance levels. This was true for both the discrete (*p* = 0.008) and cumulative (*p* =0.031) wording. For the discrete wording, ratings of performance level 5 were significantly lower than performance level 1 through 4 (*p* ≤ 0.039). Ratings of performance level 6 were also significantly lower than levels 1 through 4 (*p* ≤ 0.010). For the cumulative wording, ratings of performance level 2 were significantly lower than performance level 4 (*p* = 0.027). Ratings of performance level 6 were also significantly lower than performance level 3 through 5 (*p* ≤ 0.018).

In examining the responses, we noted the majority were actually constant across all six performance levels (i.e., the same response was selected for each performance level). Very few responses followed the expected trend of becoming more positive for the more advanced performance levels (Table 4). Additionally, the responses that were constant across performance levels tended to be at the extreme ends of the Likert scale (i.e., “very unlikely” or “very likely”) (Table 5).

**Table 4:** Percentage of participants with increasingly positive or constant responses across the levels of performance

	Discrete wording		Cumulative wording	
	Increasing	Constant	Increasing	Constant
Perceived importance	0 %	21 %	6 %	12 %
Myoelectric control	3 %	49 %	3 %	45 %
Targeted muscle reinnervation	3 %	66 %	9 %	59 %
Peripheral nerve interface	6 %	62 %	7 %	65 %
Cortical interface	6 %	71 %	3 %	81 %

**Table 5:** Percentage of constant responses across performance levels that were “very unlikely” or “very likely”

	Discrete wording		Cumulative wording	
	Very unlikely	Very likely	Very unlikely	Very likely
Myoelectric control	18 %	59 %	10 %	45 %
Targeted muscle reinnervation	52 %	22 %	17 %	39 %
Peripheral nerve interface	43 %	19 %	16 %	42 %
Cortical interface	56 %	16 %	41 %	23 %

**Fig. 5:**
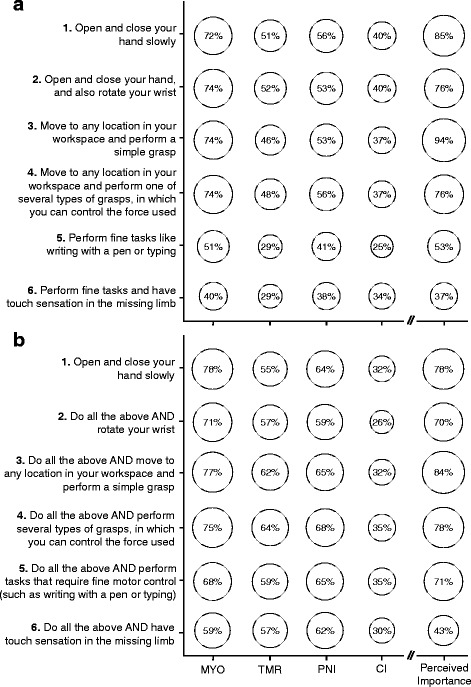
Percentage of positive responses for each technique as a function of performance level. Percentages are shown separately for the discrete (**a**) and cumulative (**b**) wording versions. Each bubble in the four leftmost columns shows the percentage of participants that responded positively (“likely” or “very likely”) to a technique at a specific performance level. Each bubble in the rightmost column shows the percentage of participants that indicated the performance level was “important” or “very important.” (MYO = myoelectric control, TMR = targeted muscle reinnervation, PNI = peripheral nerve interface, CI = cortical interface)

Participants consistently expressed the least interest in the two most advanced performance levels across all techniques (Fig. 5). A similar trend was present in participants’ responses regarding how important they considered each of the performance levels (Fig. 5). The two most advanced performance levels were considered least important for both the discrete and cumulative wording. Participants expressed the highest amount of interest in a moderately advanced performance level (level 3).

### Participant comments

**Fig. 6:**
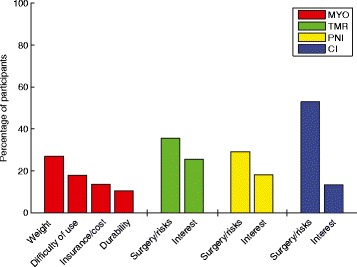
Percentage of participants who referenced common ideas in their written comments. Because participants were not required to give written comments, the values shown are percentages of the total number of comments received for each interface. Participants could reference more than one of these ideas in their comments. (MYO = myoelectric, TMR = targeted muscle reinnervation, PNI = peripheral nerve interface, CI = cortical interface)

Written comments from the participants reveal a wide variety of opinions regarding each technique (Table 6). Common concerns regarding myoelectric control included weight, difficulty of use, cost and durability (Fig. 6). Comments for the remaining three interfaces were similar. Many of the participants expressed concern about needing to undergo brain surgery in order to operate a prosthesis (Fig. 6). The inherent risk of surgery was difficult for these participants to accept, especially those who had been through extensive surgeries already. However, some participants also felt that the potential benefits would justify the surgical risks and recovery time (Fig. 6).

**Table 6:** Sample written comments from participants

Myoelectric	“The myo-electric device can be a very functional device to use, but there are limitations that come with this device that make me choose my body-powered hook more often. These limitations include durability, battery life, and not being able to get it wet. Durability is very important to me as my lifestyle is very active and I am scared to break such an expensive piece of equipment.”
	“After all of the years of wearing Ue prostheses, I don’t think the sense of touch would be important to me, but I’d try it…Compliant grasp is not that important to me - nor are other grasp patterns, simply because I’ve been able to adapt and TD into my life/ADLs…function is critical to me (not cosmesis). I also find that the weight of the prosthesis (TD) matters a lot more now.”
	“I would be willing to try this device, or others, if they were not too heavy and functioned at a reasonable level (‘reasonable’ being performing basic everyday tasks such as holding a plastic grocery bag, steadying a stationary object from movement, holding a cup or bowl, etc.).”
	“I have one. I find the myoelectric sensors difficult to use, especially if I get sweaty. The sweat changes the resistance value of my skin and causes the sensors to either become overly sensitive or not sensitive enough. This makes the prosthetic difficult to use.”
Targeted Muscle Reinnervation	“I’m willing to try anthing [*sic*] if it means a chance at better use of limb”
	“The addition of surgery and the long wait time post surgery makes this device slightly less attractive than the myoelectric device.”
	“I am satisfied with the capacities of less invasive technologies; enjoy full quality of life including functional independence, family and social relationships, leisure time activities that range from engaging in creative arts to sports. I cannot imagine risking surgical procedure that could potentially leave me with less than the magnitude that I currently enjoy.”
	“If I were to have surgery I think I would prefer to try a total limb transplant.”
	“I’m concerned about the long term effects. I’m not convinced that the medical industry knows the long term implications.”
	“At my age, surgery risk is not worth benefits.”
Peripheral Nerve Interface	“I’m concerned about the long term effects of new technology. However, I’m more open to this since it does not involve any movement of the underlying nerves.”
	“This method should be better than picking up the signals off of the skin.”
	“Very interesting process and I foresee this with development as being the mainstay of the upper extremity prosthetic field.”
	“The notion of a quick recovery is nice, as is the idea that it doesn’t rely on wires and could be used with minimal training.”
	“I’d like to see improvements with existing technologies and more studies on this before trying it myself.”
	“Sounds innovative, worth the risk.”
	“I’m not interested in having surgery performed on me in order to be able to wear/use a prosthesis.”
Cortical Interface	“Nope. Not brain surgery, thanks.”
	“This is truly incredible technology but there is nothing about this procedure that dose [*sic*] not scare me. That being said, If this is perfected and the ricks [*sic*] are reduced this could be huge for upper limb amputees. This one actually amazes me, wow!”
	“Excess risk at my age.”
	“This sounds great. I would definitely try this. I’ll be the first.”
	“It would have to be a well-documented success.”

## Discussion

Using an online survey of individuals with upper limb loss, this study examined interest in noninvasive and invasive techniques for prosthesis control. In general, participants were most interested in myoelectric control, which was the only non-invasive technique presented in the survey. It should be noted that 40 % of the participants currently use a myoelectric device, and most of the participants likely had some general familiarity with the technology. The responses to the questions about myoelectric control may consequently be biased compared to the other three techniques, which were less familiar.

Participants were generally less interested in the cortical interfaces compared to the other, less invasive techniques. Written comments provided by some of the participants suggest that the need to undergo brain surgery makes this technique unattractive. Other participants were excited about the possibilities the technology might offer them, despite the potential risks. Indeed, the fact that 39 % of the participants responded positively to cortical interfaces at any level of performance suggests that more individuals are open to this technique than might be expected.

Although responses for targeted muscle reinnervation and peripheral nerve interfaces were not statistically different, peripheral nerve interfaces were generally viewed more favorably by the participants. One possible explanation is that the post-surgery recovery time and training time are noticeably shorter for peripheral nerve interfaces than targeted muscle reinnervation.

Participants also expressed the greatest interest in more basic prosthesis features. This was true both when they were asked about their general interest in these features and whether they would use specific prosthesis technologies if these features were offered. Unexpectedly, few participants were interested in touch sensation. Prosthesis users have previously identified sensory feedback as an important design priority, but this concern was not widely shared among the individuals that were surveyed [4, 5]. Feedback from some of the participants suggests that they have become used to not having sensation in their prosthetic limb and do not view it as a priority. This is especially note-worthy since 92 % of the participants had unilateral limb loss. Having the ability to feel with an intact arm may reduce their perceived necessity of having sensation in a prosthetic arm. Nonetheless, some participants indicated that the more advanced features (fine motor control, touch sensation) would be of interest:

I understand that I can’t do certain things well, prepare a salad because of all the manipulation of vegetables and the knife, fold clothes quickly and efficiently, feel the touch of my children’s hands in mine, but I would like to see a natural looking hand that can open and close quickly to perform repetitive fine motor tasks. (Participant 95)

The fact that similar results were found with both the discrete and cumulative wording versions strengthens our findings. When the first 35 participants did not rate the advanced levels of performance highly, we thought it may have been due to misinterpretation of the questions. Since the same trend was found after changing the wording to be explicitly cumulative, we concluded that participants viewed the advanced features as less important.

With the current survey design, our results may demonstrate a “ceiling effect” for the advanced levels of performance. For instance, the examples of fine motor skills given in this survey (typing and writing with a pen) may not have been fully indicative of the skills that are considered important by individuals with upper limb loss. It is possible that using different examples would have prompted more positive responses from the participants (e.g. someone who prioritizes the ability to button a shirt over the ability to type or write with a pen may have responded negatively to the questions about fine motor skills). This limitation results from the fact that our survey was designed to evaluate general interest in prosthetic control techniques. In order to keep the survey brief, we could only present a limited number of features to participants. Future work should focus on determining the exact features that participants would want to have in their prosthesis in order to justify trying the more invasive interfaces. Even in this general survey, some participants indicated that they would expect their prosthesis to have greater functionality than what was presented in our six questions: “[Peripheral nerve interfaces are] not really for me, but if it were possible for me, I would do it AND I woukd [*sic*] expect that the hand would do all of the things listed above and more” (Participant 85).

Another limitation of this study is that is not possible to fully understand how each participant interpreted the technique descriptions. Before viewing the descriptions, participants were instructed to assume that the device was waterproof and appropriate for their amputation level (even though each drawing showed an individual with transradial limb loss), and that cost or medical restrictions would not prevent them from using the device (Additional file 1). We included these instructions to encourage participants to focus on the risks and capabilities of the devices, rather than any external factors. However, we received comments from some participants which suggested they did not recall these details. Therefore, it is possible that participants’ responses were influenced by their own experiences and knowledge of the techniques, rather than what was stated explicitly in the survey.

Another possible source of variability in participants’ interpretation of the descriptions is that discussion of any potential benefits was excluded. This was an intentional decision based on the current lack of empirical evidence to support the existence of specific benefits to each technique. Any discussion of potential benefits would therefore be theoretical and possibly misleading. We instead chose to be conservative in our descriptions and allow participants to infer potential benefits on their own. While we cannot know how participants interpreted the “benefits” of each technique, some participants explicitly wrote comments suggesting that they were able to infer potential benefits to the procedures. To demonstrate the variability in patient responses, we have included all participant comments as supplementary material (Additional file 2).

It should be noted that the descriptions presented here are for general *types* of technique, rather than specific devices. Accordingly, statements regarding medical procedures, training times, and potential risks are only estimates. The descriptions were also intentionally kept simple in order to accommodate participants with varying scientific backgrounds. More detailed and specific descriptions of the medical procedures, training times, and potential risks for each technique may influence participants’ responses.

Finally, the sample population for this study was self-selected and therefore may not be fully representative of the amputee community as a whole. For example, it is possible that use of an online survey favored “tech savvy” individuals. While we cannot measure this directly, we did collect demographic information on education. The educational levels of our sample population are slightly higher than the national averages (Table 3). These discrepancies may be due to the small size of our sample population. It is also possible that we excluded individuals who may not have computer or Internet access. However, 84 % of American households reported computer ownership in 2013 and 74 % reported Internet use in their home [28]. Administering the survey to patients at the University of Michigan Orthotics and Prosthetics Center (UMOPC) also made it available to individuals who otherwise may not have had computer access. Seventeen of the 104 participants were recruited at UMOPC. Additionally, there were no significant differences between our study population and a mail-based survey of 2477 individuals with upperlimb loss in the United States [6] in age, gender or prevalence of transradial limb loss. While there was a significant difference in prevalence of limb loss due to trauma (*p* = 0.02), trauma was the most commonly reported cause of limb loss for both studies. Similarly, other survey studies have reported trauma as the most common cause of limb loss [29–31].

## Conclusions

An online survey was used to evaluate general interest in noninvasive and invasive prosthesis control techniques. Participants were generally most interested in a noninvasive technique (myoelectric control) and least interested in a highly invasive technique (cortical interfaces). Common concerns about myoelectric control were weight, cost, durability, and difficulty of use, while the most common concern about the invasive techniques was surgical risk. Participants expressed greater interest in basic prosthesis features (i.e., opening and closing the hand slowly), as opposed to advanced features like fine motor control and touch sensation. Further study on a larger population is warranted in order to investigate the relationship between demographic factors and interest in novel prosthesis technologies. It would be interesting to know whether participants’ interest could be predicted by factors such as age, level of amputation, or satisfaction with functional abilities. This survey can be used as a basis for larger studies to explore these important questions. The results of these investigations may be used to inform the development of future prosthetic technologies that are appealing to individuals with upper limb loss.

## Additional files

Additional file 1:
**Complete descriptions of the four techniques.** Participants viewed these drawings and descriptions prior to answering questions about each technique. Additional file 2:
**All written comments received from participants.** These responses are in their original form and have not been edited for grammar or spelling. Information that could potentially reveal the participant’s identity has been redacted.
