# Supplementary material for: Surveying the interest of individuals with upper limb loss in novel prosthetic control techniques
Source: J Neuroeng Rehabil. 2015 Jun 13;12:53. doi: 10.1186/s12984-015-0044-2 (PMC4465617; doi:10.1186/s12984-015-0044-2)

Participants viewed these instructions prior to reading the technique descriptions:

The next series of questions will ask you about whether you would be willing to try new prosthetic devices or undergo surgery if you could perform certain functions. Not all of these devices are currently available to the public.

When considering each device or procedure, please:

- Focus on the capabilities of the device and risks of the procedure, rather than the potential monetary cost.
- Assume the device is appropriate for your level of amputation (even if the picture does not show your level of amputation).
- Assume you have no medical restrictions that would prevent you from using the device.
- Assume the device is waterproof and looks similar to a real arm.

Description of myoelectric control:

Myoelectric Device Controlled by Muscles in the Arm

Device Description: This device has sensors embedded in the socket of the prosthesis that touch your skin. The sensors detect electric signals that are produced when you contract your muscles. These electric signals are used to control movement of the prosthesis.

Medical Procedure: This device requires no surgery to use.

Training Time: About 3 months.

Potential Risks: There is no risk associated with using this device.

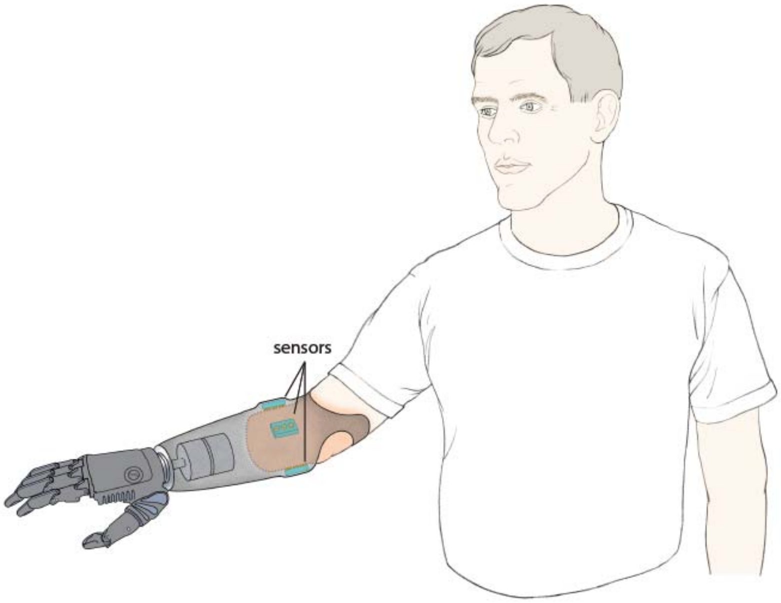

Description of targeted muscle reinnervation:

Surgery that Moves Nerves into Different Muscles

Device Description: This device operates like the myoelectric device described previously, except that you will need to have a surgical procedure before you can use it. In this procedure, the nerves that originally controlled your amputated limb are moved and connected to muscles that have not been amputated. As a result, these muscles will contract if you attempt to move your missing limb. Sensors in the prosthesis detect electric signals from these contractions and these signals are used to control movement of the prosthesis.

Medical Procedure: The nerves are moved with a surgical procedure that requires making incisions in the arm. This procedure requires a hospital stay of about 1-3 days.

The incision area will heal in about 2 weeks and become unnoticeable. After the surgery, you must wait 5-6 months to allow the nerves to regrow before you can begin training with the device.

Training Time: About 1 month.

Potential Risks: There is about a 2-3% risk of a minor medical problem (such as bruising) and a small risk of a major complication.

*** This device is not currently available to the public.*

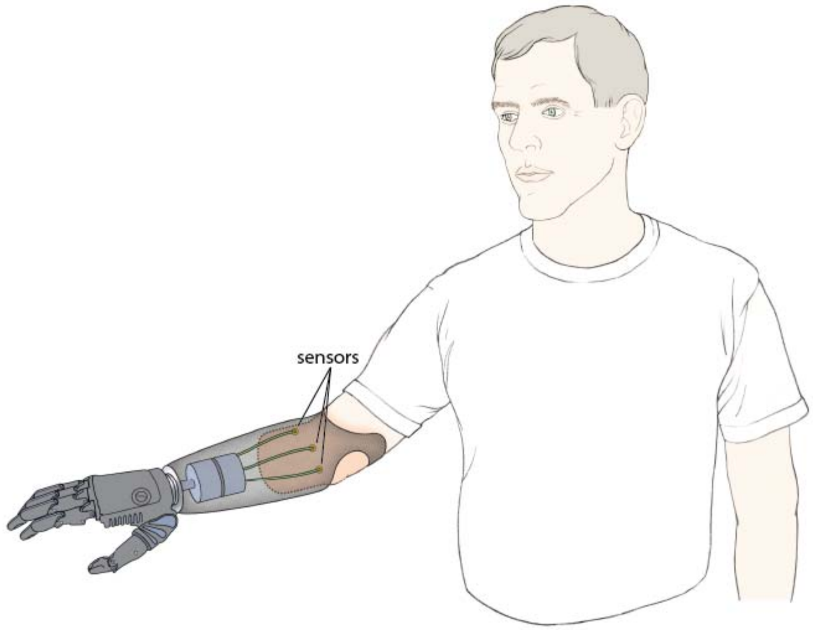

Description of peripheral nerve interfaces:

Wireless Device Implanted in the Arm

Device Description: This device is an array of 16 small sensors implanted in the arm. During a surgical procedure, small pieces of muscle are connected to the nerves that originally controlled your amputated limb. Sensors are then wrapped around these nerves and muscles. The sensors detect electric signals that are produced if you attempt to move your missing limb. These electric signals are used to control movement of the prosthesis.

Medical Procedure: This device is put in with a surgical procedure that requires opening incisions on the arm. This procedure requires a hospital stay of about 1 day. The incision area will heal in about 2 weeks and become unnoticeable.

Training Time: About 1 week.

Potential Risks: There is about a 2-3% risk of a minor medical problem (such as bruising or infection) and a small risk of a major complication. There is a small risk that additional surgery would be required to repair breakages in the system.

** *This device is not currently available to the public.*

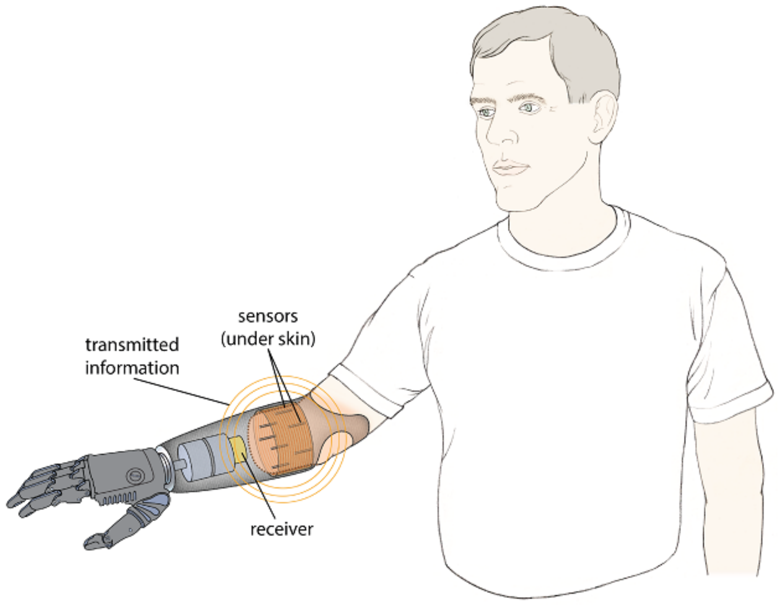

Description of cortical interfaces:

Wireless Device Implanted in the Brain

Device Description: This device is an array of 100 tiny sensors in a package about the size of a baby aspirin. The sensors are placed 1.5 mm into the surface of the brain. The device senses brain cells firing and transmits this information to a prosthetic limb.

Medical Procedure: This device is put into the brain with a surgical procedure that requires opening the skull. Before surgery, hair around the incision area must be shaved. This procedure requires a hospital stay of about 3 days. The incision area will heal in about 2 weeks and the hair will regrow, making it unnoticeable.

Training Time: About 1 week.

Potential Risks: There is about a 3-5% risk of a minor problem (such as post-operative confusion) and a 1% risk of a major complication (such as a bleed or infection).

** *This device is not currently available to the public.*

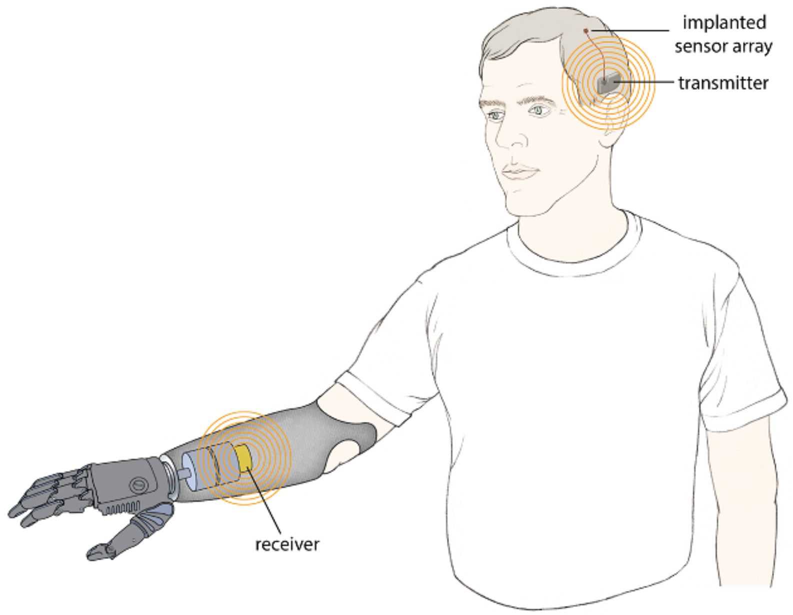

Supplement: Additional file 1: — Complete descriptions of the four techniques. Participants viewed these drawings and descriptions prior to answering questions about each technique. [file 12984_2015_44_MOESM1_ESM.pdf]
